# Promoting Roles of Melatonin in Adventitious Root Development of *Solanum lycopersicum* L. by Regulating Auxin and Nitric Oxide Signaling

**DOI:** 10.3389/fpls.2016.00718

**Published:** 2016-05-25

**Authors:** Dan Wen, Biao Gong, Shasha Sun, Shiqi Liu, Xiufeng Wang, Min Wei, Fengjuan Yang, Yan Li, Qinghua Shi

**Affiliations:** State Key Laboratory of Crop Biology, Key Laboratory of Biology and Genetic Improvement of Horticultural Crops (Huanghuai Region, Ministry of Agriculture), College of Horticulture Science and Engineering, Shandong Agricultural UniversityTaian, China

**Keywords:** adventitious root, melatonin, nitric oxide, auxin, tomato

## Abstract

Melatonin (MT) plays integral roles in regulating several biological processes including plant growth, seed germination, flowering, senescence, and stress responses. This study investigated the effects of MT on adventitious root formation (ARF) of de-rooted tomato seedlings. Exogenous MT positively or negatively influenced ARF, which was dependent on the concentration of MT application. In the present experiment, 50 μM MT showed the best effect on inducing ARF. Interestingly, exogenous MT promoted the accumulation of endogenous nitric oxide (NO) by down-regulating the expression of *S*-nitrosoglutathione reductase (*GSNOR*). To determine the interaction of MT and NO in ARF, MT synthesis inhibitor *p*-chlorophenylalanine, NO scavenger 2-(4-carboxyphenyl)-4,4,5,5-tetramethylimidazoline-1-oxyl-3-oxide potassium salt as well as *GSNOR*-overexpression plants with low NO levels were used. The function of MT was removed by NO scavenger or *GSNOR*-overexpression plants. However, application of MT synthesis inhibitor did little to abolish the function of NO. These results indicate that NO, as a downstream signal, was involved in the MT-induced ARF. Concentrations of indole-3-acetic acid and indole-3-butyric acid, as well as the expression of several genes related to the auxin signaling pathway (*PIN1, PIN3, PIN7, IAA19,* and *IAA24*), showed that MT influenced auxin transport and signal transduction as well as auxin accumulation through the NO signaling pathway. Collectively, these strongly suggest that elevated NO levels resulting from inhibited GSNOR activity and auxin signaling were involved in the MT-induced ARF in tomato plants. This can be applied in basic research and breeding.

## Introduction

Melatonin (*N*-acetyl-5-methoxytryptamine) was discovered in the bovine pineal gland in 1958 (Lerner et al., 1958), and MT was identified in plants by Dubbels et al. (1995). Up to now, MT has been extensively found in the plant kingdom at concentrations usually ranging from picograms to nanograms per gram of tissue (Fan et al., 2015; Hardeland, 2016), which influences photosynthesis (Arnao and Hernández-Ruiz, 2015), organ development (Arnao and Hernández-Ruiz, 2014), root system architecture (Pelagio-Flores et al., 2012; Zhang et al., 2014), senescence (Byeon et al., 2012; Wang et al., 2013a,b; Shi et al., 2015b), defense (Weeda et al., 2014), and stress response (Pape and Lüning, 2006; Posmyk et al., 2009; Byeon and Back, 2014; Kostopoulou et al., 2015; Liu et al., 2015b; Zhang et al., 2015) in plants. With the interest in roles of MT in plants, reports of MT in plants have dramatically increased in recent years and it is anticipated that mechanism studies related to plant MT will flourish in the near future (Tan et al., 2012). Importantly, the chemical structure (indole derivative) and biosynthetic pathway (from tryptophan) suggest that MT may be related to auxin signaling (Park, 2011; Shi et al., 2015b). The major questions that remain unanswered concern how MT functions in plants, and whether it depends on auxin signaling. Also, does MT share similar functions to auxin in plants, particularly in relation to its second messenger? If so, what acts as the second messenger? These issues stimulated us to study the relationship of MT, auxin and their second messenger based on currently available literature and emerging considerations.

Adventitious root formation (ARF) begins with redifferentiation of predetermined interfascicular parenchymatous cells in the basal region of the stem after the removal of primary root system (Pagnussat et al., 2004). ARF is frequently used in basic research and breeding as it is a simple and robust *in vitro* method for plant culture (Duclercq et al., 2011; Liu et al., 2014). ARF is evoked by auxins (López-Bucio et al., 2015) and also MT (Sarropoulou et al., 2012a). Although several researchers have studied the effects of exogenous MT on root development (Chen et al., 2009a; Sarropoulou et al., 2012b), not much is known about the molecular mechanism associated with ARF as well as the related signal transduction events. Endogenous auxin concentrations are known to be altered during the three phases of ARF: induction, initiation and extension (Yadav et al., 2011). In cucumber seedlings, a probable signaling cascade associating with nitric oxide (NO), cyclic GMP, and mitogen-activated protein kinases for auxin-induced ARF has been proposed through pharmacological approaches (Pagnussat et al., 2002, 2003, 2004). However, the mode of MT (another indole derivative of auxin) interaction with NO that results in ARF remains unclear.

Previous study indicated that NO is produced in plant tissues by two major pathways: enzymatic and non-enzymatic (Sanz et al., 2015). Three important enzymes in the enzymatic pathway of NO production have been identified that catalyze NO synthesis in plants. The first enzyme identified was nitrate reductase (NR), which usually reduces nitrate to nitrite but can also reduce nitrite to NO using NADPH as a cofactor (Sakihama et al., 2002). Another key enzyme in NO biosynthesis is *Arabidopsis thaliana* NO-associated (AtNOA1), previously described as catalyzing the conversion of L-arginine to L-citrulline (Corpas et al., 2009). More recently, *S*-nitrosoglutathione reductase (GSNOR) was confirmed to negatively regulate the NO accumulation in several plant species (Chen et al., 2009b). Additionally, NO is a signaling molecule involved in a variety of physiological processes during plant growth and development and is an important modulator of stress response and disease resistance. As the second messenger of the cGMP signaling pathway, NO plays a pivotal role in root growth and development (Hu et al., 2005). In addition, NO triggers calcium ion homeostasis (Courtois et al., 2008), phosphatidic acid accumulation (Lanteri et al., 2008), and modulates dynamic actin cytoskeleton and vesicle trafficking in a cell type-specific manner in root apices (Kasprowicz et al., 2009). Much of these effects are ascribed to the action of NO as a signaling molecule.

It was demonstrated that MT-induced sodic alkaline stress tolerance in tomato seedlings was NO dependent (Liu et al., 2015a), and temporal accumulation of endogenous NO in cucumber root was induced by exogenous indole-3-acetic acid (IAA) (Gong et al., 2014). These observations indicated a possible interaction among MT, auxin and NO, and the interaction might be involved in ARF of de-rooted tomato seedlings in the present study.

In the present study, the MT treatment affected auxin signaling events in ARF mainly by increasing NO generation. However, a reduction of MT-induced ARF was also observed in the genotype lacking NO accumulation (*GSNOR*-overexpression tomato plant) and NO scavenger [2-(4-carboxyphenyl)-4,4,5,5-tetramethylimidazoline-1-oxyl-3-oxide, potassium salt; cPTIO]-treated tomato explants. Interestingly, the auxin concentrations of apex and hypocotyl were also altered after MT addition and could be abolished by *GSNOR* overexpression or cPTIO application. Additionally, levels of auxin efflux genes (*PIN1, PIN3* and *PIN7*) and auxin signaling transduction genes (*IAA19* and *IAA24*) were significantly increased by exogenous MT application. These results strongly suggest that NO may act as a downstream signal of MT to enhance auxin signaling that induces the ARF of de-rooted tomato explants. The present study deepens understanding of the overlapping functions among MT, NO and auxin in root development as well as signaling transduction in plants.

## Materials and methods

### Plant materials, growth conditions, and rooting

Tomato seeds (*Solanum lycopersicum* L.) were sterilized in 2.5% sodium hypochlorite and washed three times for 5 min in sterile distilled water, then soaked in distilled water for 6 h at 28°C. After germination on filter paper in Petri dishes, seeds were transferred to the growth chamber filled with vermiculite maintained at 28°C with a 14-h photoperiod (photosynthetically active radiation = 200 μmol m^−2^ s^−1^). For selecting the appropriate concentration of MT, primary roots of 10-days-old seedlings were removed and tomato explants were maintained under the same conditions of temperature and photoperiod for up to 7 days with 0, 12.5, 25, 50, 75, and 100 μM MT, respectively. By the same pretreatment of tomato seedlings, 50 μM MT, 50 μM SNP (sodium nitroprusside; an NO donor), 50 μM GSNO (*S*-nitrosoglutathione; another NO donor), 200 μM cPTIO (a specific NO-scavenger), and 50 μM CPA (*p*-chlorophenylalanine; a MT synthesis inhibitor) (Park, 2011) were used, respectively.

In this study, the wild type (WT) was a self-pollinated homozygous tomato line. OE^*GSNOR*^, a transgenic tomato line with *GSNOR* overexpression (Gong et al., 2015) was used as material with lower concentration of NO.

### Detection of NO

The NO formation was measured using the fluorescent dye diaminofluorescein-FM diacetate (DAF-FM DA; Sigma, Stockholm, Sweden). Hypocotyls were placed in 1 ml of buffer solution (10 mM Tris-HCl, pH 7.2) and then incubated for 20 min at room temperature with 1 ml of DAF-FM DA at a final concentration of 5 μM in loading buffer (10 mM Tris-HCl, pH 7.2). The incubation solutions were then pipetted off and hypocotyls were washed three times with fresh loading buffer to remove excess fluorochrome. After being washed, the hypocotyls were mounted on a microscope slide in the same medium for examination with a confocal laser scanning microscope system, using standard filters and collection modalities for DAF-FM DA green fluorescence (excitation 495 nm; emission 515 nm). The pixel intensities of fluorescence images, acquired using a confocal microscope, were determined using ImageJ software (Schneider et al., 2012).

Nitric Oxide content was determined according to the method of luminol-H_2_O_2_ chemiluminescent (Kikuchi et al., 1993), with some modifications in plant (Gao et al., 2009). Briefly, NO generation was estimated by a luminol-H_2_O_2_ chemiluminescence method. This method is based on the specific chemiluminescence reaction of luminol with peroxynitrite, a stronger oxidizing species than H_2_O_2_ that can be produced by the reaction of NO with H_2_O_2_, in alkaline carbonate buffer. Samples were ground in an ice bath with a mortar and pestle at a ratio of 200 mg fresh weight per mL of cold deoxygenated water (freshly prepared by boiling distilled deionized water for 1 h). The extracts were centrifuged at 12,000 × g for 20 min at 4°C and the supernatant fraction was immediately used for chemiluminescence detection using a computerized BPCL ultra-weak chemiluminescence analyzer (Institute of Biophysics, Academia Sinica, China) at one-second intervals by a photon-counting method. NO concentration was expressed as chemiluminescence counts per gram of fresh weight (counts g^−1^ FW).

### Quantification of MT

MT was extracted using the acetone-methanol method (Pape and Lüning, 2006). Briefly, 1 g of samples of apex or hypocotyl from different treatments were ground in liquid nitrogen, and then transferred to 5 ml of extraction mixture (acetone:methanol:water = 89:10:1) and homogenized extensively on ice, and the homogenate was centrifuged at 4500 × g for 5 min at 4°C. The supernatant was moved to a new centrifuge tube containing 0.5 ml of 1% trichloroacetic acid for protein precipitation. After centrifugation at 12 000 × g for 10 min at 4°C, the extract was used for quantification of MT using an ELISA Kit (EK-DSM; Buhlmann Laboratories AG, Schonenbuch, Switzerland) according to the manufacturer's instructions.

### Semi-quantitative PCR and real-time quantitative PCR analyses

Total RNA was extracted from plant using the TRIzol method according to the supplier's instructions (Invitrogen, Carlsbad, CA, USA). DNase was used during RNA extraction to reduce DNA contamination. cDNA synthesis was performed according to standard procedures of a Revert Aid First Strand cDNA synthesis kit (Fermentas, Ontario, Canada). Tomato β -*actin* gene was used as the internal control for quantification of transcripts. Semi-quantitative PCR and real-time quantitative PCR (RT-qPCR) were performed per primer pair (Supplementary Table 1). Semi-quantitative PCR was carried out using the following program: an initial denaturation at 94°C for 3 min, followed by 24–30 cycles of 94°C for 30 s, T_*m*_ – 5°C for 30 s and 72°C for 1 min, and a final extension at 72°C for 10 min, and PCR products were detected by 1% agarose gel in 1 × TAE with ethidium bromide. Gels were visualized under short wave ultraviolet (UV) light in a UVP Biospectrum AC Imaging System GelDoc station and photographed using a digital camera. RT-qPCR used an aliquot of cDNA (1/500), Power SYBR Green PCR Master Mix (Applied Biosystems, Calif., USA), and 200 nM of each primer on an ABI Prism 7900 HT machine. Data were analyzed using SDS 2.500 software (ABI), and relative expression was calculated using the comparative cycle threshold method with normalization of data to the geometric average of the internal control genes (Pfaffl, 2001).

### IAA content measurement

Harvested samples of 20 different plants were rinsed with distilled water, blotted dry, freeze-dried, and then frozen in liquid nitrogen immediately after excision, ground and immediately weighed. The tissue samples were homogenized in a methanol solution. The extract was passed through a C_18_ column on a solid phase extractor and the eluate was collected. Then 1 ml of methanol was continuously added to the C_18_ column. IAA in the extracts was quantified by high performance liquid chromatography (Waters 515, Massachusetts, USA) with a UV detector (254 nm) and a quantitative tube that automatically and accurately controlled the set volume injected. The operating conditions were a Hypersil reversed phase C_18_ column (μ Bondapak, 250 mm × 4.6 mm i.d.), a mobile phase of methanol:water (4:1; v/v), a flow rate of 0.6 ml min^−1^, an injection volume of 20 μl and a temperature of 25°C. Under these conditions, the retention time for IAA was 4 min.

### Statistical analysis

Data were subjected to analysis of variance (ANOVA) using SPSS 22.0 software (IBM SPSS Corp., Armon, NY, USA). Differences between treatments were determined by Duncan's multiple range test at *p* < 0.05 level. Data were plotted using Origin 8.6 software (OriginLab, Northampton, MA, USA). Data are presented as the mean ± standard deviation of three replicates.

## Results

### Dose-dependent curves for ARF in response to exogenous MT

**Figure 1:**
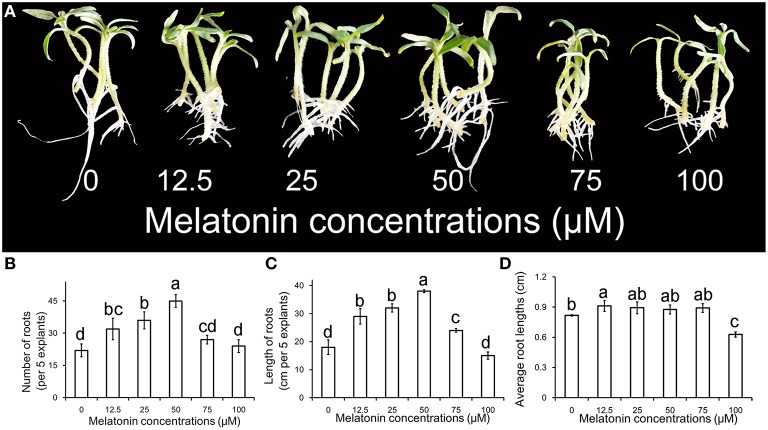
**Adventitious root formation in response to exogenous MT of different concentrations. (A)** The primary root system was removed from hypocotyls of 10-days-old germinated tomato. Explants were incubated with 0, 12.5, 25, 50, 75, or 100 μM MT. Photographs were taken after 6 days of treatments. **(B)** Root numbers in different concentrations of exogenous MT. **(C)** Root lengths in different concentrations of exogenous MT. **(D)** Average root lengths in different concentrations of exogenous MT. These are expressed as means of five replicates ± standard deviation (SD), and different letters indicate significant difference (*p* < 0.05).

The phenotype of ARF treated with different concentrations of MT is shown in Figure 1A. The primary root system was removed from hypocotyls of 10-days-old tomato seedlings and explants were incubated for 6 days with 0, 12.5, 25, 50, 75, and 100 μM MT, respectively. Compared with no MT treatment, treatment with exogenous MT significantly enhanced the numbers of adventitious roots for the concentrations applied (*p* < 0.05). The number of adventitious root peaked for 50 μM, and then declined with increasing MT concentration in Figure 1B. However, single roots became shorter with MT treatment that resulted in greater total length of adventitious roots. Interestingly, the effect of MT on ARF appeared to be promoting at low concentration and repressing at high concentration (Figures 1C,D). This characteristic makes MT seem like a novel phytohormone, which is of great significance to plants. According to our comprehensive estimations for these parameters and phenotype, the most powerful concentration of MT (50 μM) for induction of adventitious root was used for further studies.

### Effects of exogenous MT on endogenous NO metabolism

**Figure 2:**
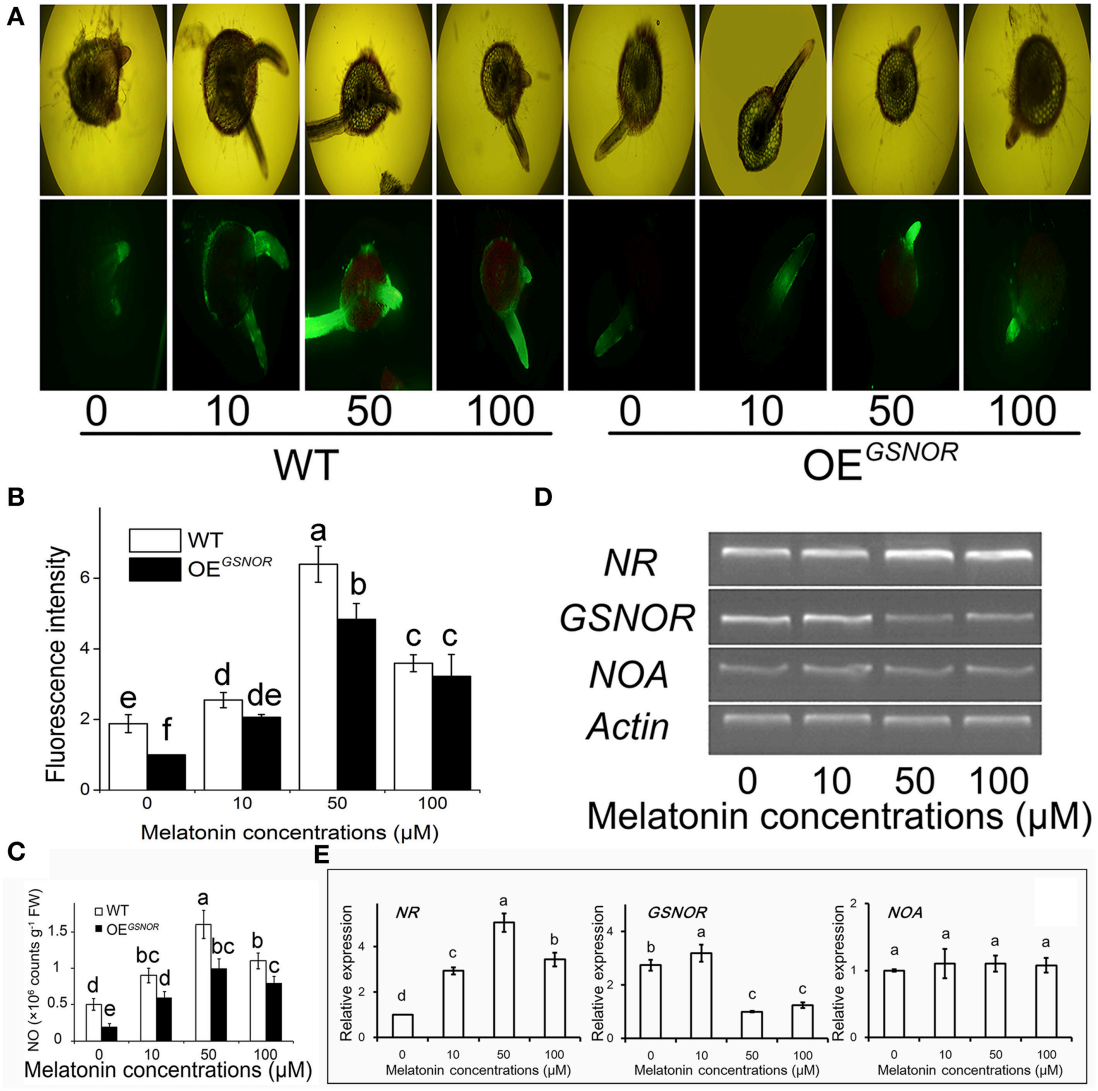
**Effects of exogenous MT on endogenous NO metabolism. (A)** Detection of contents of MT-induced NO by fluorescence staining. 4,5-Diamino-fluorescein diacetate fluorescence detected in a cross section from 3 mm distance to the tip of the hypocotyls, where new meristematic tissue and adventitious root formed. Photographs were taken after 3 days of treatment. WT indicates WT tomato and OE^*GSNOR*^ indicates tomato overexpressing *GSNOR.* **(B)** NO content in different treatments. **(C)** Relative expression of NO metabolism related genes. Values are expressed as means of three replicates ± SD, and different letters indicate significant difference (*p* < 0.05). **(D)** Fluorescence intensity of NO expressed as arbitrary units (A.U.) using ImageJ software. The same letter above a column indicates no difference at *p* < 0.05. **(E)** Expressions of *NR, GSNOR* and *NOA* measured by RT-QPCR. Values are expressed as means of three replicates ± SD, and different letters indicate significant difference (*p* < 0.05).

To explore the role of NO in MT signaling, we examined MT-induced NO production in hypocotyls during root formation. The NO content was determined at 3 days of treatment of the de-rooted tomato hypocotyls, where new adventitious roots regenerated. With 50 μM MT treatment, the NO content of WT reached the highest level (Figure 2C) as well as relative fluorescence intensity (Figure 2B). Meanwhile, different treatments were loaded with the permeable NO-sensitive fluorophore DAF-FM DA. There was increasing NO-associated fluorescence in cross sections of MT-treated hypocotyls, especially in the pericycle, epidermis, and adventitious roots (Figure 2A). The intensity of fluorescence was increased by MT treatment and peaked for 50 μM, and then markedly decreased with increasing MT concentration. In contrast to WT, OE^*GSNOR*^ tomato showed lower endogenous NO levels under the same conditions, and the no-MT treatment showed particularly faint fluorescence. In addition, although the layers of cortex increased moderately, the total cell number increased drastically after MT treatment. All of these implied that exogenous MT may have activated endogenous NO signaling, which may have initially stimulated differentiation of the pericycle which has great potential to control the exchange of signal substances through the epidermis as the main communication channel. In order to check the mechanism of MT regulating NO, the expression of *NR, GSNOR,* and *NOA* were determined by semi-quantitative PCR at 2 days of MT treatment (Figure 2D) and RT-qPCR data were also produced (Figure 2E). In relation to *actin*, the expression of *NR* was up-regulated by MT treatment, with the highest intensity for 50 μM MT. In contrast, *GSNOR* expression was down-regulated by MT treatment. *NOA* expression showed no significant differences for the different MT concentrations.

### Effects of exogenous MT on the endogenous auxin

**Figure 3:**
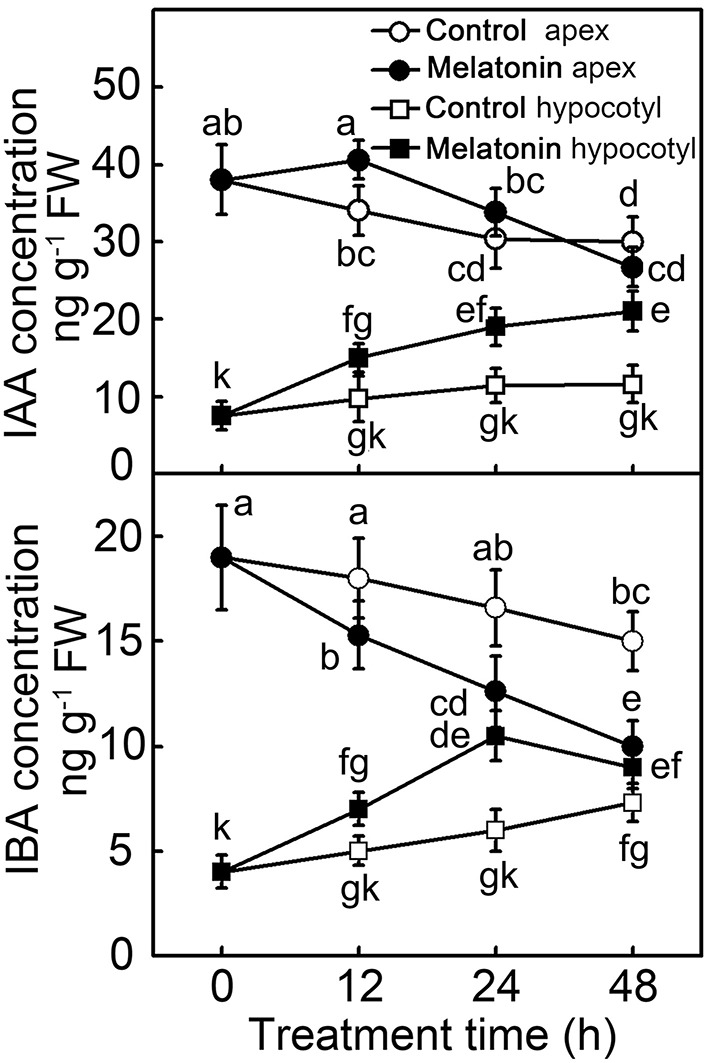
**Concentration of IAA and IBA during the MT-induced adventitious root development**. The primary system was removed from hypocotyls of 10-days-old tomato seedlings. Explants were incubated with water (Control) and 50 μM MT (MT). Data represent the means of five replicates ± SD, and different letters indicate significant difference (*p* < 0.05).

The formation of adventitious roots is a complex genetic trait regulated by environmental and endogenous factors, among which the phytohormone auxin plays an essential role. Because exogenous MT showed a similar function to auxin in the induction of adventitious roots, concentrations of IAA and indole-3-butytric acid (IBA) were determined (Figure 3). Compared with hypocotyl, concentrations of both IAA and IBA were higher in the apex. In the 48 h of post-excision, the IAA concentration in hypocotyl increased, especially in the MT treatment, which doubled the IAA concentration. Showing an opposite trend, the IAA concentration in the apex of control decreased throughout the 48 h of rooting process; while MT treatment led to IAA increasing in the first 12 h, and then decreasing until 48 h. Similarly, with or without MT application, the IBA concentration showed an increasing and decreasing tendency in hypocotyl and apex, respectively. At 24 h, the IBA concentration in MT-treated hypocotyl reached its highest level, and then slightly decreased until 48 h of treatment. All these differences indicated that IAA and IBA contents as well as their distribution were regulated in de-rooted tomato seedlings by exogenous MT.

### Effects of exogenous NO on endogenous MT accumulation

**Figure 4:**
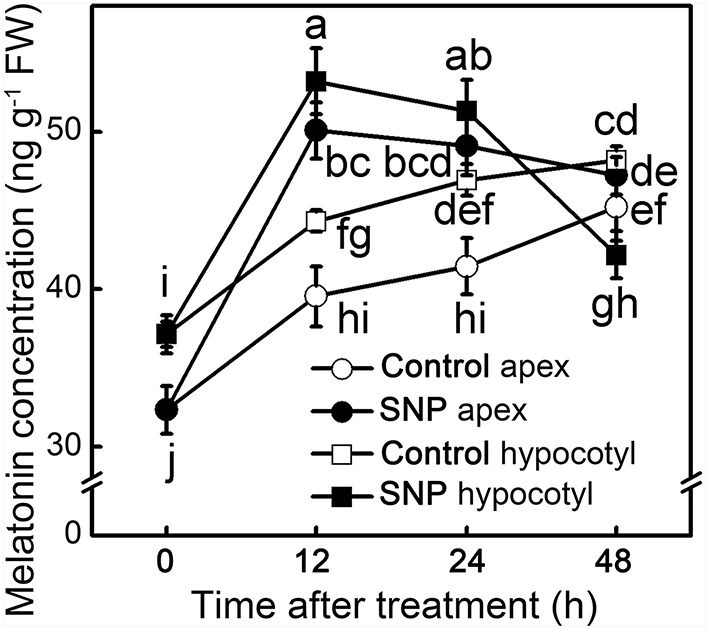
**MT concentration during the NO-induced adventitious root development**. The primary root system was removed from hypocotyls of 10-days-old tomato seedlings. Explants were incubated with water (Control) and 50 μM SNP (SNP). Data represent the means of five replicates ± SD, and different letters indicate significant difference (*p* < 0.05).

MT concentration in different parts was dynamically monitored to elucidate the effect of exogenous NO on endogenous MT. After removing the primary root system, endogenous MT kept increasing in both apex and hypocotyl of control seedlings. The application of SNP significantly increased the endogenous MT concentration at 12 h of treatment (*p* < 0.05), and then its concentration declined (Figure 4). MT induced by NO may function as signaling at early stages and be used afterwards in ARF of the hypocotyl. Before 12 h, SNP induced large increases in MT concentration both in hypocotyl and apex, indicating that NO could play a positive role in response to MT in the MT-promoted ARF signaling pathway.

### Interaction of MT and NO involves the signaling pathway of inducing ARF

**Figure 5:**
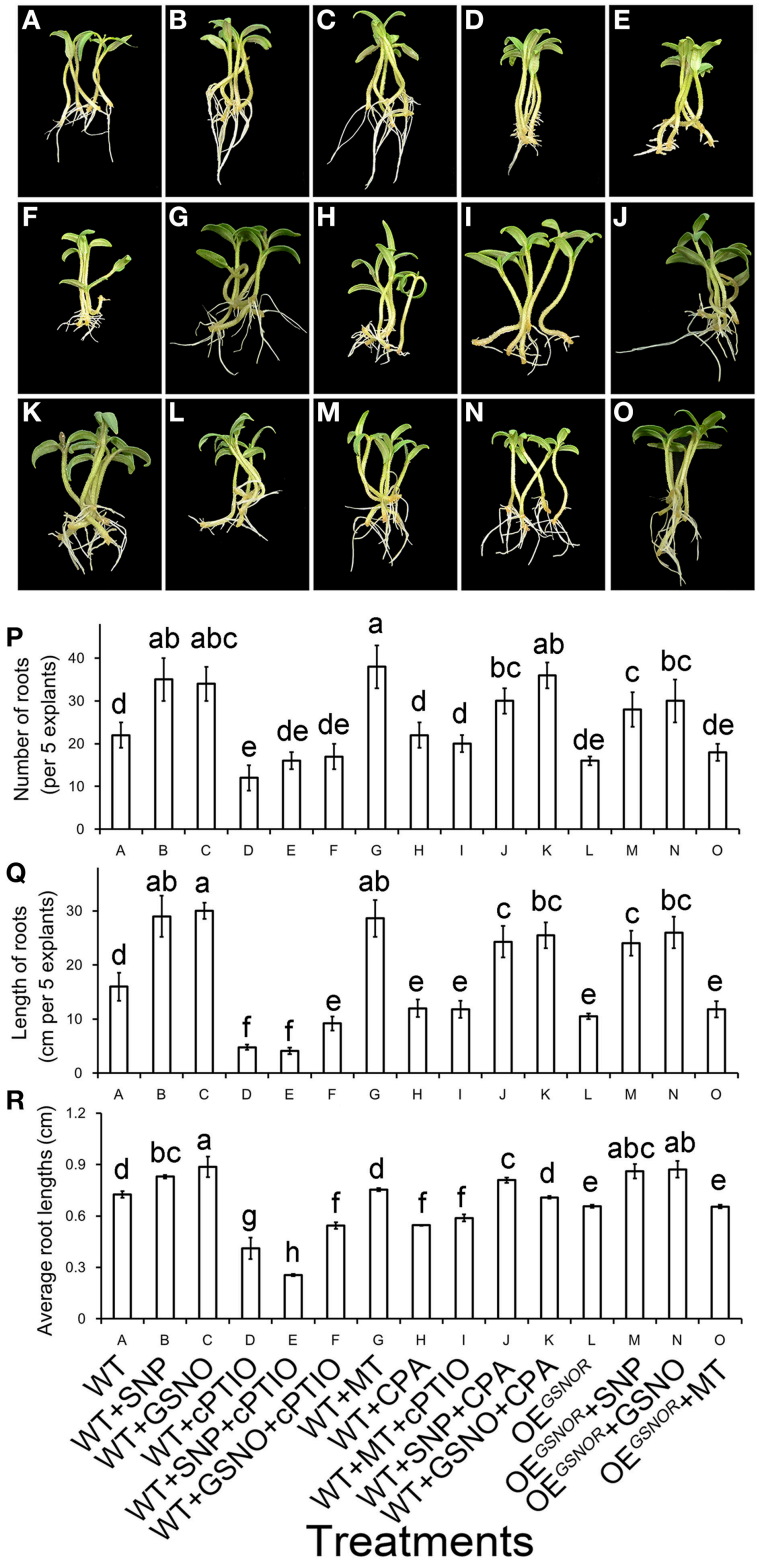
**Interactive effect of MT and NO on adventitious root formation**. The primary system was removed from hypocotyls of 10-days-old germinated tomato. **(A)** WT; **(B)** WT + SNP; **(C)** WT + GSNO; **(D)** WT + cPTIO; **(E)** WT + SNP + cPTIO; **(F)** WT + GSNO + cPTIO; **(G)** WT + MT; **(H)** WT + CPA; **(I)** WT + MT + cPTIO; **(J)** WT + SNP + CPA; **(K)** WT + GSNO + CPA; **(L)** OE^*GSNOR*^; **(M)** OE^*GSNOR*^ + SNP; **(N)** OE^*GSNOR*^ + GSNO; **(O)** OE^*GSNOR*^ + MT. Photographs were taken after 5 days of treatment. **(P)** Root numbers in different treatments. **(Q)** Root lengths in different treatments. **(R)** Average root lengths in different treatments. These are expressed as means of five replicates ± SD, and different letters indicate significant difference (*p* < 0.05).

Pharmacological and genetic methods were used to clarify the interaction of MT and NO in the process of promoting ARF. Application of NO significantly promoted ARF (*p* < 0.05) (Figures 5A–C), while the function of NO was removed by cPTIO (Figures 5D,L). Similarly, adventitious roots increased in numbers and total length with MT treatment (Figure 5G), and greatly decreased with CPA (Figure 5H). Interestingly, after treating WT with MT + cPTIO (Figure 5I), the rooting situation was much better compared to treatment with only cPTIO (Figure 5D). However, the situation of treating WT with MT + cPTIO was even worse than WT treated with SNP + CPA (Figure 5J) or GSNO + CPA (Figure 5K). This indicated that NO was a downstream signal of MT in ARF. The phenotype for OE^*GSNOR*^ with MT treatment (Figure 5O) was very similar to that for WT with MT + cPTIO treatment (Figure 5I), and both these treatments got lower score for ARF than WT with NO + CPA treatment (Figures 5J,K). Root numbers, lengths and average root lengths are corresponding with the phenotype (Figures 5P–R). From the genetic aspect, this suggested that NO played an important role in ARF.

### Role of IAA and NO in the process of ARF regulation by MT

**Figure 6:**
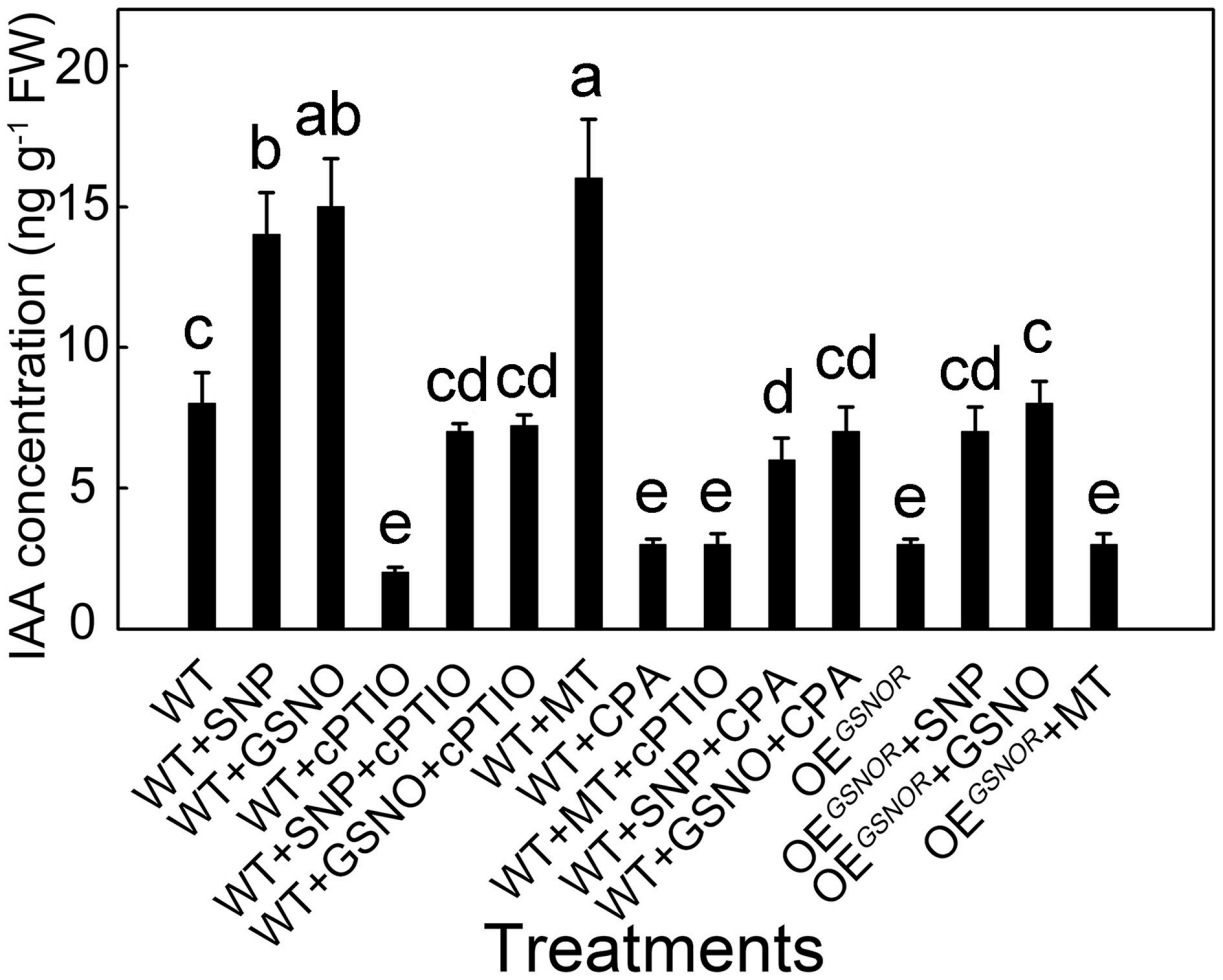
**IAA concentration with different treatments during adventitious root development in the hypocotyl sampled at 12 h**. Values are expressed as means of five replicates ± SD, and different letters indicate significant difference (*p* < 0.05).

To determine the effects of different treatments on auxin accumulation and ARF in the explants, the levels of IAA were monitored in the hypocotyl of different combined treatments. At 12 h, IAA concentrations in the MT treatment and control were significantly different (Figure 3) and at this time the SNP treatment resulted in a large difference in MT concentration (Figure 4). MT treatment markedly increased the IAA concentration (*p* < 0.05; Figure 6), and similar results were obtained in exogenous NO treatments in which SNP and GSNO were used as NO donors. Whereas the accumulation of IAA induced by MT was dramatically abolished by cPTIO application, in contrast, the CPA had a much lower effect on IAA accumulation induced by GSNO and SNP. Similarly, cPTIO also decreased IAA levels in other treatments including WT, WT with SNP and WT with GSNO. OE^*GSNOR*^ tomato seedlings were used as lower NO material, and showed much lower IAA accumulation compared to WT. The SNP and GSNO application significantly increased IAA level in OE^*GSNOR*^, while MT had no significant effect on IAA accumulation in WT.

### Genes expression related with auxin signaling in ARF

**Figure 7:**
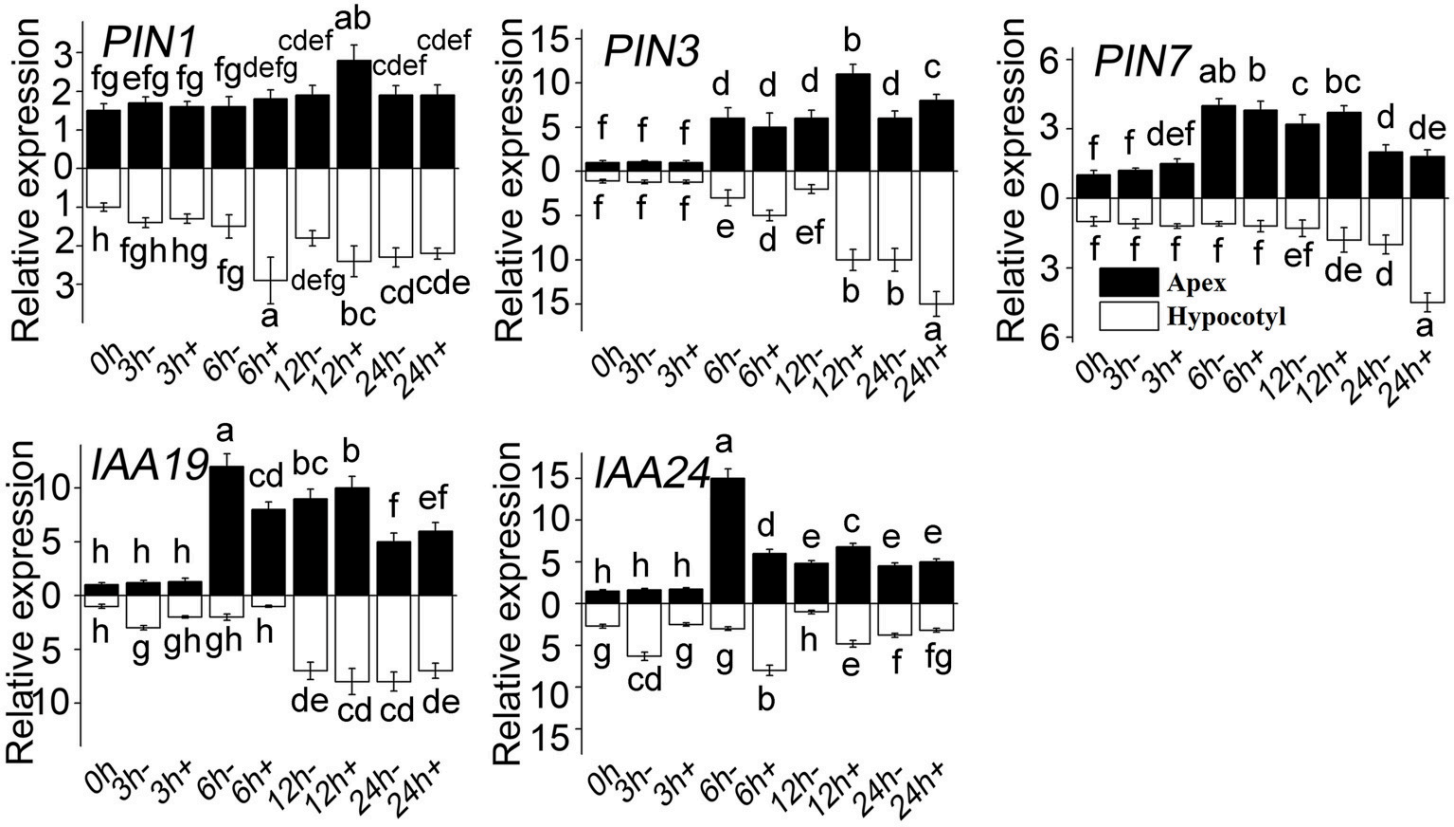
**Relative expression of genes in response to MT treatment**. RT-QPCR analyses were used to assess the apex and hypocotyl samples at 0, 3, 6, 12, and 24 h with (+) or without (-) MT treatment. Values are expressed as means of three replicates ± SD, and different letters indicate significant difference (*p* < 0.05).

It was previously demonstrated that auxin plays a vital important role in ARF. In the present study, a series of genes related to auxin signaling was analyzed firstly by Semi-quantitative PCR (Supplementary Figures 1–3). To ensure the screening results, RT-QPCR was used to detect the expression of those genes that showed more response to MT (Figure 7). The level of *PIN1, PIN3,* and *PIN7* increased both in apex and in hypocotyl treated with MT. In the early stage, the *PIN1* level in hypocotyl increased sharply, the level of *PIN3* also increased with time, and the level of *PIN7* increased at a comparatively later period. Compared with WT, *IAA19* decreased in hypocotyl at the early stage after MT treatment, and then increased. The same trend occurred for the apex, except that there was no difference at 3 h. MT treatment decreased the *IAA24* level at 3 h and increased it at 6 h in hypocotyl.

## Discussion

The present study provides evidence that NO acts as a positive modulator of the MT and auxin signaling pathway in ARF. Previous study indicated that MT induced the accumulation of IAA in *Brassica juncea* roots (Chen et al., 2009a). In the present study, we also provided evidence that the MT-induced tomato ARF depended on the MT function in regulating auxin metabolism. Furthermore, in this process, NO, as a downstream signal of MT, played an important role. Shi et al. (2015a) reported that MT-induced NO production was responsible for innate immunity response of *Arabidopsis* against *Pst* DC3000 infection. However, the interactions between MT and NO, as well as other important molecules, especially related to auxin signaling, in ARF are largely unknown.

Hernández-Ruiz et al. (2005) showed that MT inhibited root elongation in some monocots, canary grass, and oats, even at very low concentrations of 10 μM. Herein, we observed that exogenous MT increased root growth in 10-days-old tomato explants at low concentrations, but inhibited root growth at higher concentrations, in accordance with a previous report (Chen et al., 2009a). It is known that MT has the same precursor as and similar physiological functions to auxin. The latter molecule also has bimodal effects on plant growth: promoting growth at low concentrations and inhibiting growth at high concentrations (Ljung, 2014; Laplaze et al., 2015). So, we speculated that low MT concentration promoting the ARF was probably due to the regulation of auxin accumulation or distribution. In the present study, 50 μM MT had the maximal positive effect on ARF of tomato explants. Endogenous IAA and IBA levels of both apex and hypocotyl indicated that stimulation of ARF by 50 μM MT was due to its function in stimulating IAA accumulation and acropetal auxin transport (Figure 3). IBA, the second most abundant natural auxin, generally participated in ARF because of its stability when exposed to light compared to IAA. It was shown that ABCG36 and ABCG37 appeared to efflux IBA rather than IAA (Strader and Bartel, 2009). Interestingly, it was demonstrated that IBA was converted into IAA and was proposed to be active only after its conversion (Pacurar et al., 2014). Hence, the transportation and accumulation of IBA was also important for ARF. For the reason of increased IAA and IBA in hypocotyl were only a little more than reduced IAA and IBA in apex. Thus, MT's effect on ARF of de-rooted hypocotyls seemed to involve mechanisms mainly depending on acropetal auxin transport. However, the specific mechanism remains unknown.

As an important gaseous molecule and secondary messenger, NO-mediated root development and the association between auxin and NO are well-documented (Abu-Abied et al., 2012; Terrile et al., 2012). Application of exogenous NO was suggested to promote ARF in *Arabidopsis* (Campos-Cuevas et al., 2008), cucumber (Pagnussat et al., 2004), and marigold (Liao et al., 2012), while inhibitors of NO generation attenuated the ARF in *Arabidopsis* and other plant species (Abu-Abied et al., 2012; Terrile et al., 2012). The crosstalk between auxin and NO in root development was determined in previous studies, which led us to determine the relationship between MT and NO. As a new insight, the analysis of NO fluorescence probes provides strong evidence that MT stimulated the NO accumulation through NR and GSNOR pathways (Figure 2). More importantly, the organizational location of MT-induced NO was shown to mainly accumulate in the cambium and phloem of hypocotyls as well as new adventitious roots, implying that NO was involved in the MT-mediated ARF process. Moreover, compared with WT explants, less accumulation of NO as well as a weaker capacity for ARF in *GSNOR*-overexpression explants also provided support for our observations. Collision with the traditional view in animal, several previous studies suggested that MT reduces nitric oxide synthase activity in hypothalamus (Bettahi et al., 1996), cerebellum (Pozo et al., 1994), and liver (Taysi et al., 2003) of rat. However, Thakor et al. (2010) showed that MT activated vasodilatation and increased conductance in the umbilical vascular bed by stimulating NO synthesis. There are several mechanisms via which MT could increase NO bioavailability. MT can prolong the action of NO by inhibiting cGMP-phosphodiesterase in animal (Ting et al., 2000). Recently, MT was also reported to induce NO generation in *Arabidopsis* (Shi et al., 2015a) and tomato (Liu et al., 2015a). These differences of MT and NO relationship may be explained in part by dose-dependent and regional effects of the indole amine. At the same time, it also indicates a complex relationship between MT and NO in plants.

The present study provided several lines of evidence that NO, as a downstream signal, was involved in MT-induced ARF of tomato. Firstly, MT-induced ARF was accompanied by up-regulating *NR* and down-regulating *GSNOR*, which elevated endogenous NO levels in de-rooted hypocotyls (Figure 2). Secondly, scavenging of NO by cPTIO or inhibiting NO generation by overexpressing *GSNOR* abolished MT-induced ARF (Figure 5). Thirdly, exogenous NO also induced ARF, which was not abolished by CPA (Figure 5). In addition, the temporal changes in auxin accumulation (Figure 6) and transcript levels of genes involved in auxin signal transport (Figure 7) were consistent with the role of NO in MT-induced ARF. However, we also observed that NO increased the levels of MT in both apex and hypocotyls (Figure 4), indicating a possible NO feedback to MT biosynthesis in tomato plants. The nature of the relationship between MT and NO is still highly controversial: MT and its precursors may act as scavengers of NO (Noda et al., 1999) but MT also induces NO accumulation. Here, we conjecture that low amounts of NO are produced after application of MT, which acts as a signaling molecule, playing a critical role in the ARF process. These provide a new insight into the ability of MT to work together with other signaling pathways to regulate plant development.

In addition, MT altered the expression of a large number of auxin-related genes. The polar localization of PIN proteins is responsible for auxin transport and disruption, which directly affect IAA transport to regulate the ARF process (Yadav et al., 2010). A previous study indicated that *PIN1* promoted the auxin lateral efflux from the hypocotyl vasculature to the pericycle founder cells in ARF (Della Rovere et al., 2013), which suggests that MT-induced *PIN1* expression was beneficial for ARF in our study (Figure 7). *PIN3* is redirected toward one side of the columella cells and determines the direction of auxin flux, which leads to asymmetric auxin accumulation and differential growth (Friml et al., 2002). *PIN3* redistributes the auxin toward locating in columella cells, where adventitious roots and accumulated NO were detected (Figure 2A). MT also increased the abundance of *PIN7* transcripts with a three- to five-fold enhancement in hypocotyl (Figure 7). This suggests that *PIN7* may make more contribution to cell proliferation and elongation rather than differentiation. Auxin/IAA proteins are transcriptional regulators that mediate many aspects of plant responses to auxin (Wang et al., 2005). We examined the abundance of these transcripts with distinct spatial and temporal expression patterns, and found that *IAA19* and *IAA24* were significantly changed.

**Figure 8:**
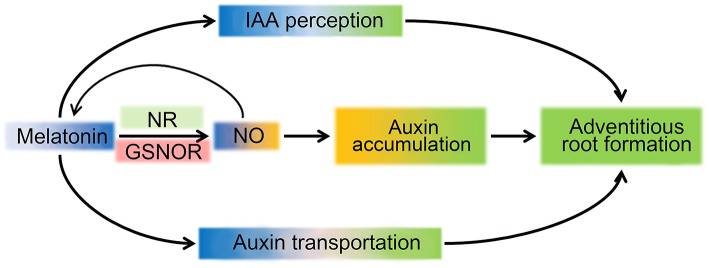
**Schematic illustration for MT signaling induced ARF**. MT triggers NO accumulation by up-regulating expression of *NR* and down-regulating *GSNOR*. In turn, NO feedback controls the accumulation of MT. The accumulation of auxin could be induced by NO to improve the ARF process. In parallel, perception and transport of the auxin signal are also present, leading to ARF in the MT signaling pathway.

Based on aforementioned results and analysis, a schematic illustration for a possible mechanism of MT regulating ARF in tomato was produced (Figure 8). MT triggers NO accumulation by up-regulation of *NR* expression and down-regulation of *GSNOR* expression, and NO increased IAA accumulation, which combined with transportation and distribution of auxin regulated by MT, play important roles in ARF. However, research on MT signals in plants is just at the beginning, and more work is needed to gain a more accurate understanding of the MT signal pathway in ARF regulation.

## Author contributions

Conceived and designed the research: QS, BG, and DW. Performed the research: SS and YL. Analyzed the data: BG and DW. Contributed reagents/materials/analysis tools: SL, XW, MW, and FY. Wrote the first draft of the manuscript: DW. Improved the first draft of the manuscript: QS and BG. All authors have read and approved this manuscript.

### Conflict of interest statement

The authors declare that the research was conducted in the absence of any commercial or financial relationships that could be construed as a potential conflict of interest.

## Supplementary material

Supplementary Figure 1 **Semi-quantitative PCR expression profiles of individual *SlPIN* genes**. The relative mRNA level of individual *SlPIN* genes was normalized with respect to the housekeeping gene, *actin*, in apex, and hypocotyl with different treatment times.

Supplementary Figure 2 **Semi-quantitative PCR expression profiles of individual *SlIAA* genes**. The relative mRNA level of individual *SlIAA* genes was normalized with respect to the housekeeping gene, *actin*, in apex, and hypocotyl with different treatment times.

Supplementary Figure 3 **Semi-quantitative PCR expression profiles of individual *SlAFB* genes**. The relative mRNA level of individual *SlAFB* genes was normalized with respect to the housekeeping gene, *actin*, in apex, and hypocotyl with different treatment times.

Supplementary Table 1 **Primers for semi-quantitative PCR and real-time quantitative PCR**.

## References

[B1] Abu-Abied M. Szwerdszarf D. Mordehaev I. Levy A. Belausov E. Yaniv Y.. (2012). Microarray analysis revealed upregulation of nitrate reductase in juvenile cuttings of *Eucalyptus grandis*, which correlated with increased nitric oxide production and adventitious root formation. Plant J. 71, 787–799. 10.1111/j.1365-313X.2012.05032.x 22519851

[B2] Arnao M. B. Hernández-Ruiz J. (2014). Melatonin: plant growth regulator and/or biostimulator during stress? Trends Plant Sci. 19, 789–797. 10.1016/j.tplants.2014.07.006 25156541

[B3] Arnao M. B. Hernández-Ruiz J. (2015). Functions of melatonin in plants: a review. J. Pineal Res. 59, 133–150. 10.1111/jpi.12253 26094813

[B4] Bettahi I. Pozo D. Osuna C. Reiter R. J. Acuña-Castroviejo D. Guerrero J. M. (1996). Melatonin reduces nitric oxide synthase activity in rat hypothalamus. J. Pineal Res. 20, 205–210. 10.1111/j.1600-079X.1996.tb00260.x 8836954

[B5] Byeon Y. Back K. (2014). Melatonin synthesis in rice seedlings *in vivo* is enhanced at high temperatures and under dark conditions due to increased serotonin *N*-acetyltransferase and *N*-acetylserotonin methyltransferase activities. J. Pineal Res. 56, 189–195. 10.1111/jpi.12111 24313332

[B6] Byeon Y. Park S. Kim Y. S. Park D. H. Lee S. Back K. (2012). Light-regulated melatonin biosynthesis in rice during the senescence process in detached leaves. J. Pineal Res. 53, 107–111. 10.1111/j.1600-079X.2012.00976.x 22289080

[B7] Campos-Cuevas J. C. Pelagio-Flores R. Raya-González J. Méndez-Bravo A. Ortiz-Castro R. López-Bucio J. (2008). Tissue culture of *Arabidopsis thaliana* explants reveals a stimulatory effect of alkamides on adventitious root formation and nitric oxide accumulation. Plant Sci. 174, 165–173. 10.1016/j.plantsci.2007.11.003

[B8] Chen Q. Qi W. Reiter R. J. Wei W. Wang B. (2009a). Exogenously applied melatonin stimulates root growth and raises endogenous indoleacetic acid in roots of etiolated seedlings of *Brassica juncea*. J. Plant Physiol. 166, 324–328. 10.1016/j.jplph.2008.06.002 18706737

[B9] Chen R. Sun S. Wang C. Li Y. Liang Y. An F.. (2009b). The *Arabidopsis PARAQUAT RESISTANT2* gene encodes an *S*-nitrosoglutathione reductase that is a key regulator of cell death. Cell Res. 19, 1377–1387. 10.1038/cr.2009.117 19806166

[B10] Corpas F. J. Palma J. M. Del Río L. A. Barroso J. B. (2009). Evidence supporting the existence of l-arginine-dependent nitric oxide synthase activity in plants. New Phytol. 184, 9–14. 10.1111/j.1469-8137.2009.02989.x 19659743

[B11] Courtois C. Besson A. Dahan J. Bourque S. Dobrowolska G. Pugin A.. (2008). Nitric oxide signalling in plants: interplays with Ca^2+^ and protein kinases. J. Exp. Bot. 59, 155–163. 10.1093/jxb/erm197 18212029

[B12] Della Rovere F. Fattorini L. D'Angeli S. Veloccia A. Falasca G. Altamura M. (2013). Auxin and cytokinin control formation of the quiescent centre in the adventitious root apex of *arabidopsis*. Ann. Bot. 112, 1395–1407 10.1093/aob/mct215 24061489 PMC3806543

[B13] Dubbels R. Reiter R. Klenke E. Goebel A. Schnakenberg E. Ehlers C.. (1995). Melatonin in edible plants identified by radioimmunoassay and by high performance liquid chromatography-mass spectrometry. J. Pineal Res. 18, 28–31. 10.1111/j.1600-079X.1995.tb00136.x 7776176

[B14] Duclercq J. Sangwan-Norreel B. Catterou M. Sangwan R. S. (2011). *De novo* shoot organogenesis: from art to science. Trends Plant Sci. 16, 597–606. 10.1016/j.tplants.2011.08.004 21907610

[B15] Fan J. Hu Z. Xie Y. Chan Z. Chen K. Amomobo E.. (2015). Alleviation of cold damage to photosystem II and metabolisms by melatonin in *Bermudagrass*. Front. Plant Sci. 6:925. 10.3389/fpls.2015.00925 26579171 PMC4630300

[B16] Friml J. Wiśniewska J. Benková E. Mendgen K. Palme K. (2002). Lateral relocation of auxin efflux regulator PIN3 mediates tropism in *Arabidopsis*. Nature 415, 806–809. 10.1038/415806a 11845211

[B17] Gao H. J. Yang H. Q. Wang J. X. (2009). Arginine metabolism in roots and leaves of apple (*Malus domestica* Borkh.): the tissue-specific formation of both nitric oxide and polyamines. Sci. Hortic. 119, 147–152. 10.1016/j.scienta.2008.07.034

[B18] Gong B. Miao L. Kong W. Bai J. G. Wang X. Wei M.. (2014). Nitric oxide, as a downstream signal, plays vital role in auxin induced cucumber tolerance to sodic alkaline stress. Plant Physiol. Biochem. 83, 258–266. 10.1016/j.plaphy.2014.08.004 25194776

[B19] Gong B. Wen D. Wang X. Wei M. Yang F. Li Y.. (2015). *S*-Nitrosoglutathione reductase-modulated redox signaling controls sodic alkaline stress responses in *Solanum lycopersicum* L. Plant Cell Physiol. 56, 790–802. 10.1093/pcp/pcv007 25634962

[B20] Hardeland R. (2016). Melatonin in plants-diversity of levels and multiplicity of functions. Front. Plant Sci. 7:198. 10.3389/fpls.2016.00198 26925091 PMC4759497

[B21] Hernández-Ruiz J. Cano A. Arnao M. B. (2005). Melatonin acts as a growth-stimulating compound in some monocot species. J. Pineal Res. 39, 137–142. 10.1111/j.1600-079X.2005.00226.x 16098090

[B22] Hu X. Neill S. J. Tang Z. Cai W. (2005). Nitric oxide mediates gravitropic bending in soybean roots. Plant Physiol. 137, 663–670. 10.1104/pp.104.054494 15681661 PMC1065366

[B23] Kasprowicz A. Szuba A. Volkmann D. Baluška F. Wojtaszek P. (2009). Nitric oxide modulates dynamic actin cytoskeleton and vesicle trafficking in a cell type-specific manner in root apices. J. Exp. Bot. 60, 1605–1617. 10.1093/jxb/erp033 19261922 PMC2671617

[B24] Kikuchi K. Nagano T. Hayakawa H. Hirata Y. Hirobe M. (1993). Detection of nitric oxide production from a perfused organ by a luminol-hydrogen peroxide system. Anal. Chem. 65, 1794–1799. 10.1021/ac00061a025 8368532

[B25] Kostopoulou Z. Therios I. Roumeliotis E. Kanellis A. K. Molassioltis A. (2015). Melatonin combined with ascorbic acid provides salt adaptation in *Citrus aurantium* L. seed. Plant Physiol. Biochem. 86, 155–165. 10.1016/j.plaphy.2014.11.021 25500452

[B26] Lanteri M. L. Laxalt A. M. Lamattina L. (2008). Nitric oxide triggers phosphatidic acid accumulation via phospholipase D during auxin-induced adventitious root formation in cucumber. Plant Physiol. 147, 188–198. 10.1104/pp.107.111815 18375601 PMC2330318

[B27] Laplaze L. Lucas M. Champion A. (2015). Rhizobial root hair infection requires auxin signaling. Trends Plant Sci. 20, 332–334. 10.1016/j.tplants.2015.04.004 25920666

[B28] Lerner A. B. Case J. D. Takahashi Y. Lee T. H. Mori W. (1958). Isolation of melatonin, the pineal gland factor that lightens melanocytes. J. Am. Chem. Soc. 80, 2587 10.1021/ja01543a060

[B29] Liao W. B. Huang G. B. Yu J. H. Zhang M. L. (2012). Nitric oxide and hydrogen peroxide alleviate drought stress in marigold explants and promote its adventitious root development. Plant Physiol. Biochem. 58, 6–15. 10.1016/j.plaphy.2012.06.012 22771430

[B30] Liu J. Sheng L. Xu Y. Li J. Yang Z. Huang H.. (2014). *WOX11* and *12* are involved in the first-step cell fate transition during de novo root organogenesis in *Arabidopsis*. Plant Cell. 26, 1081–1093. 10.1105/tpc.114.122887 24642937 PMC4001370

[B31] Liu N. Gong B. Jin Z. Wang X. Wei M. Yang F.. (2015a). Sodic alkaline stress mitigation by exogenous melatonin in tomato needs nitric oxide as a downstream signal. J. Plant Physiol. 186, 68–77. 10.1016/j.jplph.2015.07.012 26412100

[B32] Liu N. Jin Z. Wang S. Gong B. Wen D. Wang X. (2015b). Sodic alkaline stress mitigation with exogenous melatonin involves reactive oxygen metabolism and ion homeostasis in tomato. Sci. Hortic. 181, 18–25. 10.1016/j.scienta.2014.10.049

[B33] Ljung K. (2014). Auxin-a simple compound with a profound effect on plant development. Physiol Plant. 151, 1–2. 10.1111/ppl.12184

[B34] López-Bucio J. Ortiz-Castro R. Ruíz-Herrera L. F. Juárez C. V. Hernández-Madrigal F. Carreón-Abud Y.. (2015). Chromate induces adventitious root formation via auxin signalling and SOLITARY-ROOT/IAA14 gene function in *Arabidopsis thaliana*. Biometals 28, 353–365. 10.1007/s10534-015-9838-8 25702099

[B35] Noda Y. Mori A. Liburdy R. Packer L. (1999). Melatonin and its precursors scavenge nitric oxide. J. Pineal Res. 27, 159–163. 10.1111/j.1600-079X.1999.tb00611.x 10535765

[B36] Pacurar D. I. Perrone I. Bellini C. (2014). Auxin is a central player in the hormone cross-talks that control adventitious rooting. Physiol Plant. 151, 83–96. 10.1111/ppl.12171 24547793

[B37] Pagnussat G. C. Lanteri M. L. Lamattina L. (2003). Nitric oxide and cyclic GMP are messengers in the indole acetic acid-induced adventitious rooting process. Plant Physiol. 132, 1241–1248. 10.1104/pp.103.022228 12857806 PMC167064

[B38] Pagnussat G. C. Lanteri M. L. Lombardo M. C. Lamattina L. (2004). Nitric oxide mediates the indole acetic acid induction activation of a mitogen-activated protein kinase cascade involved in adventitious root development. Plant Physiol. 135, 279–286. 10.1104/pp.103.038554 15122018 PMC429373

[B39] Pagnussat G. C. Simontacchi M. Puntarulo S. Lamattina L. (2002). Nitric oxide is required for root organogenesis. Plant Physiol. 129, 954–956. 10.1104/pp.004036 12114551 PMC1540240

[B40] Pape C. Lüning K. (2006). Quantification of melatonin in phototrophic organisms. J. Pineal Res. 41, 157–165. 10.1111/j.1600-079X.2006.00348.x 16879322

[B41] Park W. J. (2011). Melatonin as an endogenous plant regulatory signal: debates and perspectives. J. Plant Biol. 54, 143–149. 10.1007/s12374-011-9159-6

[B42] Pelagio-Flores R. Muñoz-Parra E. Ortiz-Castro R. López-Bucio J. (2012). Melatonin regulates *Arabidopsis* root system architecture likely acting independently of auxin signaling. J. Pineal Res. 53, 279–288. 10.1111/j.1600-079X.2012.00996.x 22507071

[B43] Pfaffl M. W. (2001). A new mathematical model for relative quantification in real-time RT-PCR. Nucleic Acids Res. 29, 2001–2007. 10.1093/nar/29.9.e45 11328886 PMC55695

[B44] Posmyk M. M. Bałabusta M. Wieczorek M. Sliwinska E. Janas K. (2009). Melatonin applied to cucumber (*Cucumis sativus* L.) seeds improves germination during chilling stress. J. Pineal Res. 46, 214–223. 10.1111/j.1600-079X.2008.00652.x 19141087

[B45] Pozo D. Reiter R. J. Calvo J. R. Guerrero J. M. (1994). Physiological concentrations of melatonin inhibit nitric oxide synthase in rat cerebellum. Life Sci. 55, PL455–PL460. 10.1016/0024-3205(94)00532-X 7527477

[B46] Sakihama Y. Nakamura S. Yamasaki H. (2002). Nitric oxide production mediated by nitrate reductase in the green alga *Chlamydomonas reinhardtii*: an alternative NO production pathway in photosynthetic organisms. Plant Cell Physiol. 43, 290–297. 10.1093/pcp/pcf034 11917083

[B47] Sanz L. Albertos P. Mateos I. Sánchez-Vicente I. Lechón T. Fernández-Marcos M.. (2015). Nitric oxide (NO) and phytohormones crosstalk during early plant development. J. Exp. Bot. 66, 2857–2868. 10.1093/jxb/erv213 25954048

[B48] Sarropoulou V. Dimassi-Theriou K. Therios I. Koukourikou-Petridou M. (2012b). Melatonin enhances root regeneration, photosynthetic pigments, biomass, total carbohydrates and proline content in the cherry rootstock PHL-C (*Prunus avium*× *Prunus cerasus*). Plant Physiol. Biochem. 61, 162–168. 10.1016/j.plaphy.2012.10.001 23127522

[B49] Sarropoulou V. N. Therios I. N. Dimassi-Theriou K. N. (2012a). Melatonin promotes adventitious root regeneration in in vitro shoot tip explants of the commercial sweet cherry rootstocks CAB-6P (*Prunus cerasus* L.), Gisela 6 (*P. cerasus*× *P. canescens*), and M × M 60 (*P. avium*× *P. mahaleb*). J. Pineal Res. 52, 38–46. 10.1111/j.1600-079X.2011.00914.x 21749439

[B50] Schneider C. A. Rasband W. S. Eliceiri K. W. (2012). NIH Image to ImageJ: 25 years of image analysis. Nat. Methods 9, 671–675. 10.1038/nmeth.2089 22930834 PMC5554542

[B51] Shi H. Chen Y. Tan D. X. Reiter R. J. Chan Z. He C. (2015a). Melatonin induces nitric oxide and the potential mechanisms relate to innate immunity against bacterial pathogen infection in *Arabidopsis*. J. Pineal Res. 59, 102–108. 10.1111/jpi.12244 25960153

[B52] Shi H. Reiter R. J. Tan D. X. Chan Z. (2015b). *INDOLE-3-ACETIC ACID INDUCIBLE 17* positively modulates natural leaf senescence through melatonin-mediated pathway in *Arabidopsis*. J. Pineal Res. 58, 26–33. 10.1111/jpi.12188 25324183

[B53] Strader L. C. Bartel B. (2009). The *Arabidopsis* PLEIOTROPIC DRUG RESISTANCE8/ABCG36 ATP binding cassette transporter modulates sensitivity to the auxin precursor indole-3-butyric acid. Plant Cell. 21, 1992–2007. 10.1105/tpc.109.065821 19648296 PMC2729616

[B54] Tan D. X. Hardeland R. Manchester L. C. Korkmaz A. Ma S. Rosales-Corral S.. (2012). Functional roles of melatonin in plants, and perspectives in nutritional and agricultural science. J. Exp. Bot. 63, 577–597. 10.1093/jxb/err256 22016420

[B55] Taysi S. Koc M. Büyükokuroğlu M. E. Altınkaynak K. Şahin Y. N. (2003). Melatonin reduces lipid peroxidation and nitric oxide during irradiation-induced oxidative injury in the rat liver. J. Pineal Res. 34, 173–177. 10.1034/j.1600-079X.2003.00024.x 12614476

[B56] Terrile M. C. París R. Calderón-Villalobos L. I. Iglesias M. J. Lamattina L. Estelle M.. (2012). Nitric oxide influences auxin signaling through S-nitrosylation of the *Arabidopsis* TRANSPORT INHIBITOR RESPONSE 1 auxin receptor. Plant J. 70, 492–500. 10.1111/j.1365-313X.2011.04885.x 22171938 PMC3324642

[B57] Thakor A. S. Herrera E. A. Serón-Ferré M. Giussani D. A. (2010). Melatonin and vitamin C increase umbilical blood flow via nitric oxide-dependent mechanisms. J. Pineal Res. 49, 399–406. 10.1111/j.1600-079X.2010.00813.x 20958954

[B58] Ting N. Thambyraja A. Sugden D. Scalbert E. Delagrange P. Wilson V. (2000). Pharmacological studies on the inhibitory action of melatonin and putative melatonin analogues on porcine vascular smooth muscle. Naunyn Schmiedebergs Arch. Pharmacol. 361, 327–333. 10.1007/s002109900198 10731047

[B59] Wang H. Jones B. Li Z. Frasse P. Delalande C. Regad F.. (2005). The tomato *Aux/IAA* transcription factor *IAA9* is involved in fruit development and leaf morphogenesis. Plant Cell. 17, 2676–2692. 10.1105/tpc.105.033415 16126837 PMC1242265

[B60] Wang P. Sun X. Chang C. Feng F. Liang D. Cheng L.. (2013a). Delay in leaf senescence of *Malus hupehensis* by long-term melatonin application is associated with its regulation of metabolic status and protein degradation. J. Pineal Res. 55, 424–434. 10.1111/jpi.12091 24103092

[B61] Wang P. Sun X. Li C. Wei Z. Liang D. Ma F. (2013b). Long-term exogenous application of melatonin delays drought-induced leaf senescence in apple. J. Pineal Res. 54, 292–302. 10.1111/jpi.12017 23106234

[B62] Weeda S. Zhang N. Zhao X. Ndip G. Guo Y. Buck G. A.. (2014). *Arabidopsis* transcriptome analysis reveals key roles of melatonin in plant defense systems. PLoS ONE 9:e93462. 10.1371/journal.pone.0093462 24682084 PMC3969325

[B63] Yadav S. David A. Bhatla S. C. (2010). Nitric oxide modulates specific steps of auxin-induced adventitious rooting in sunflower. Plant Signal Behav. 5, 1163–1166. 10.4161/psb.5.10.12159 20948300 PMC3115341

[B64] Yadav S. David A. Bhatla S. C. (2011). Nitric oxide accumulation and actin distribution during auxin-induced adventitious root development in sunflower. Sci. Hortic. 129, 159–166. 10.1016/j.scienta.2011.03.030

[B65] Zhang N. Sun Q. Zhang H. Cao Y. Weeda S. Ren S.. (2015). Roles of melatonin in abiotic stress resistance in plants. J. Exp. Bot. 66, 647–656. 10.1093/jxb/eru336 25124318

[B66] Zhang N. Zhang H. J. Zhao B. Sun Q. Q. Cao Y. Y. Li R.. (2014). The RNA-seq approach to discriminate gene expression profiles in response to melatonin on cucumber lateral root formation. J. Pineal Res. 56, 39–50. 10.1111/jpi.12095 24102657

