# Supplementary figures and images for: Promoting Roles of Melatonin in Adventitious Root Development of Solanum lycopersicum L. by Regulating Auxin and Nitric Oxide Signaling
Source: Front Plant Sci. 2016 May 25;7:718. doi: 10.3389/fpls.2016.00718 (PMC4879336; doi:10.3389/fpls.2016.00718)

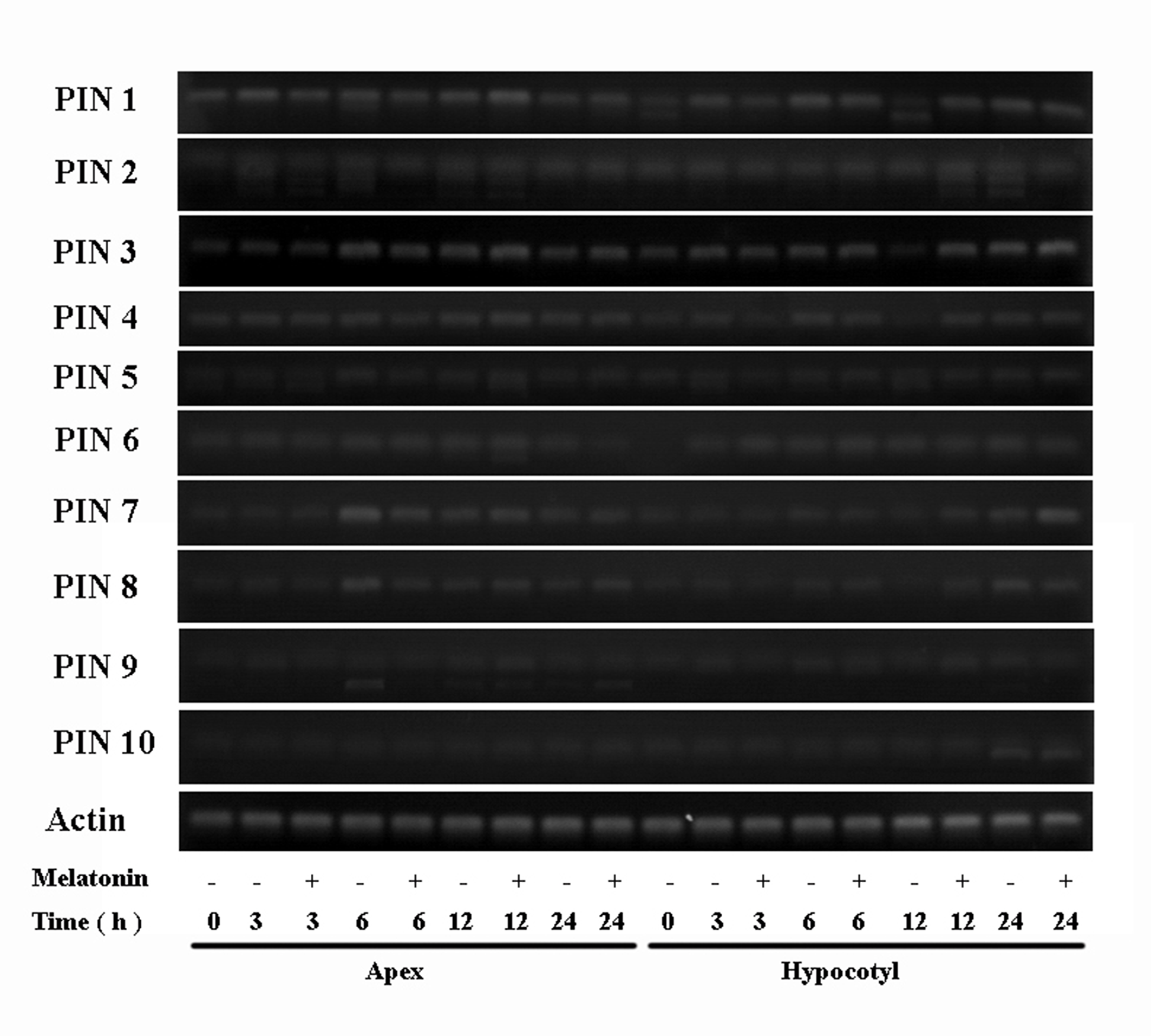

Supplement: Supplementary Figure 1 — Semi-quantitative PCR expression profiles of individual SlPIN genes. The relative mRNA level of individual SlPIN genes was normalized with respect to the housekeeping gene, actin, in apex, and hypocotyl with different treatment times. [file Image1.TIF]

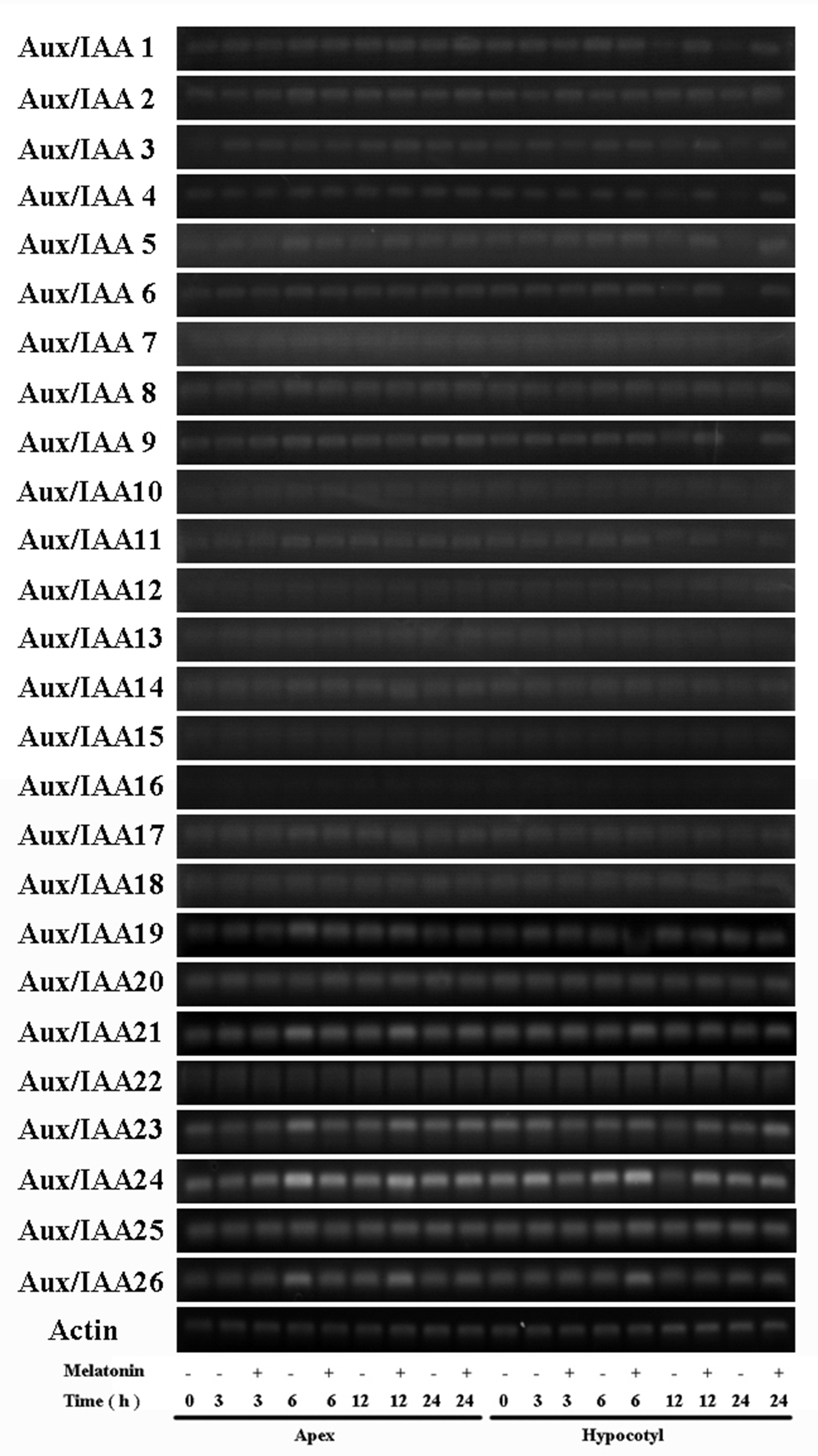

Supplement: Supplementary Figure 2 — Semi-quantitative PCR expression profiles of individual SlIAA genes. The relative mRNA level of individual SlIAA genes was normalized with respect to the housekeeping gene, actin, in apex, and hypocotyl with different treatment times. [file Image2.TIF]

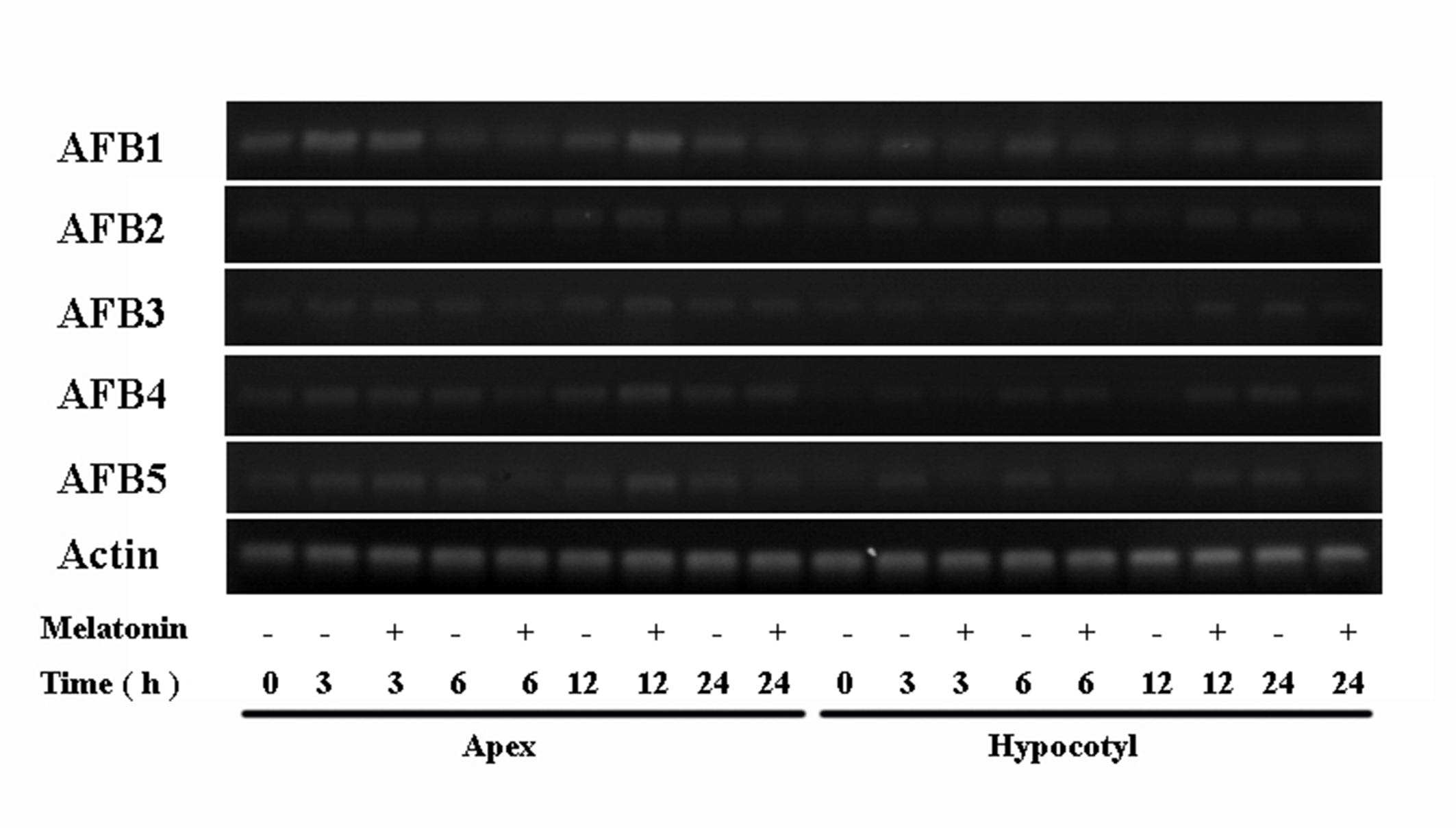

Supplement: Supplementary Figure 3 — Semi-quantitative PCR expression profiles of individual SlAFB genes. The relative mRNA level of individual SlAFB genes was normalized with respect to the housekeeping gene, actin, in apex, and hypocotyl with different treatment times. [file Image3.TIF]
