# Supplementary material for: Promoting Roles of Melatonin in Adventitious Root Development of Solanum lycopersicum L. by Regulating Auxin and Nitric Oxide Signaling
Source: Front Plant Sci. 2016 May 25;7:718. doi: 10.3389/fpls.2016.00718 (PMC4879336; doi:10.3389/fpls.2016.00718)
Supplement: Supplementary Table 1 — Primers for semi-quantitative PCR and real-time quantitative PCR. [file Table1.DOC]

**Supplymentary Table |** Primers for Semi-quantitative PCR and real-time Quantitative PCR

| Gene | Forward primer (5' - 3') | Reverse primer (5' - 3') | Sequence ID |
| --- | --- | --- | --- |
| *SlNR* | GAGTTCTCCCTCCACACGTT | ATGGCACGGAGTTGTAATGA | Solyc11g013810.1.1 |
| *SlGSNOR* | GGAGTTATGATGAACGACCG | GCCAAAGACGGCAACAAT | Solyc09g064370.2.1 |
| *SlNOA* | CGATGCTTGTTCCCTTTGTT | TCTTGCTTCTGCTGACCTGT | Solyc02g079400.2.1 |
| *SlACTIN* | GGTGTGATGGTGGGTATGG | GCTGACAATTCCGTGCTC | Solyc04g011500.2.1 |
| *SlPIN1* | GCTGCAGGCTGGTCTAGATT | AACAATGGCAACAAAGCACA | Solyc03g118740.2.1 |
| *SlPIN2* | GTGCCAGTGCCAGTACCAGT | GACCAAATGAGTCCGATCAAA | Solyc07g006900.1.1 |
| *SlPIN3* | TTCAAAATCAATTTAGCGTGTCA | CTCAAAATCCCTCTTGTTTCG | Solyc04g007690.2.1 |
| *SlPIN4* | AAAGAGGGACCCACTGGACT | TCATAACACTAGCCGGAGGC | Solyc05g008060.2.1 |
| *SlPIN5* | ACATTGAGCTGGCATTTTGG | TCCACTACCAGCCTTTGACA | Solyc01g068410.2.1 |
| *SlPIN6* | AGATGGCAGCAATAGGGATG | GCGAAGACAAATGGAACGAT | Solyc06g059730.1.1 |
| *SlPIN7* | ATCGTTCCTTTCGTCTTTGC | GCATAAAAAGCAGTGGCAAAAT | Solyc10g080880.1.1 |
| *SlPIN8* | GGTGGGGGAAACTCTTTTCA | TTCCTTTGGGCTTCAGTTTG | Solyc02g087660.2.1 |
| *SlPIN9* | TCCTGAGGCATCACCATACA | TCATTGCTTCCAACAATCCA | Solyc10g078370.1.1 |
| *SlPIN10* | GACAGGTGGCATTTCGGACTGCC | CCAGGGGGTAATGCTGCCTGAA | Solyc04g056620.1.1 |
| *SlAFB1* | GCTCTGTCATTGTTGAGTTGTG | AAGCACCTCCAGTGATGTAAAG | Solyc02g079190.2.1 |
| *SlAFB2* | CGTGTTTGGAGTCTTTGACC | GCCTGTTCCCAGATTGGTA | Solyc04g074980.2.1 |
| *SlAFB3* | GAGAACTGGACCTGGGAGAA | TGGGAACAGCACGATTGA | Solyc06g008780.1.1 |
| *SlAFB4* | TGGACCTGGGAGAAAGTGA | TCAAATGAGGAGAGCGAGC | Solyc06g008780.2.1 |
| *SlAFB5* | TAGTGGGCATTGGCTAAGC | GGGCACAGCACGATTTATC | Solyc04g074520.2.1 |
|  |  |  |  |
|  |  |  |  |
| Gene | Forward primer (5' - 3') | Reverse primer (5' - 3') | Reference |
| *SlIAA1* | GGTTTACCAGGGAGGACAG | GGGATCTGAAATGGAGTTTG | S1 |
| *SlIAA2* | GAAGCGAGCTATGTTAAAG | CATCCGTTGTATCCATCTG | S1 |
| *SlIAA3* | GCCTGGGATAATAAACGATG | CAACTTGTGCTTTTGGTGC | S1 |
| *SlIAA4* | GGTCTATCTGATTGTTCGTCG | CAAGTCTCCTCACCTCTTTCG | S1 |
| *SlIAA5* | GCCCAAGTGTTACAAAGAC | GGCACTTGTAGAAGCAGATC | S1 |
| *SlIAA6* | GATGATGAAGGGAGAACAGG | CACTGACCAATACTAAAGCAG | S1 |
| *SlIAA7* | CCTGGTGGTGGAGGAGATC | CTTAGTTGGTGGCTTGTTGG | S1 |
| *SlIAA8* | GCTTATGGACCTTACAAATGG | CATCTCCTACAAGCATCCAGTC | S1 |
| *SlIAA9* | GCTCTAATGATCCTGCTAAG | CACCGTCAACACTAACTTTC | S1 |
| *SlIAA10* | CCTTACCTTAGGAAAGTGGAC | GAAGGAATTGAACATCTTGG | S1 |
| *SlIAA11* | GAACAGTTTTAACGGACGTG | CTTATCTGCATCCTCCAATG | S1 |
| *SlIAA12* | TATGAACCCACAACACCC | GGCTCCATCCATGCTAAC | S1 |
| *SlIAA13* | GCAACTTGTCTACAGGGATG | GAGCAAAAGAGACTAACGGC | S1 |
| *SlIAA14* | GGTCCCAAATAAGAGTGCC | CTTCCAATAGGAATACCATCC | S1 |
| *SlIAA15* | TCTTAGATTGGGTTTGCC | ACGAGGTGTTGTGGCTTC | S1 |
| *SlIAA16* | GGGCTAAGGCTATTCCAAG | CGATCTGCAAATTCGTTCC | S1 |
| *SlIAA17* | GATCAACAAGACTCCTCCC | GTGCTCCATCCATGCTAAC | S1 |
| *SlIAA18* | ATCCTTACTAACACTTTGCTCC | ACAGTAGTGTAAAGCGTCCATC | S1 |
| *SlIAA19* | ATGTCACGGTGGAGAACG | GCAATTCCAACTCCTTCC | S1 |
| *SlIAA20* | CAAATGCTCTTCCTGGTC | CGTATCCTTTTAGCCACC | S1 |
| *SlIAA21* | CATCATCAAGGTCATGCAG | CCTTCCTTCCTATTGGCAC | S1 |
| *SlIAA22* | CCAGACAAAACTACCACCC | GAGAAGCCTTGACGAGCCC | S1 |
| *SlIAA23* | CCACCAGTTCGATCATACAG | CAAATAAGGTGCTCCATCC | S1 |
| *SlIAA24* | GGGTTTTCTGAGACTGTTG | AGGTGGCTTGATTGGATC | S1 |
| *SlIAA25* | TGATGAGAAGGGAGTTGG | CCTTTTCTTTAACCGATGC | S1 |
| *SlIAA26* | CCAGATAAGAATGATTACCCAC | CTTTTACACCCTTTGGAGAAAC | S1 |
| *SlARF1* | CATGGGATATGGAACCACTTGTC | TCGAGCACGCTTATTTCTTTGA | S2 |
| *SlARF2* | CAGCCTAAGCGGCATCTTCTTA | GAAAAGAACAGAATCTCCAGCAACA | S2 |
| *SlARF3* | GTCTGAGAACTGTTGTAGGTATCCGT | CCTGTTTGCTTTTGCTCCTTTAAG | S2 |
| *SlARF4* | ATGAGCTGCGACAGGAATTAGG | ACAAGCTGCCAGCCTGATCTT | S2 |
| *SlARF5* | GTGTCGGAATAGTTGATGCTGATAC | TACACCAGCTCACCCTCTCGC | S2 |
| *SlARF6* | GATATGTCTCGGCAGCCTCCA | TAGGCTGGCCCCGGAATATA | S2 |
| *SlARF7* | AATTGGGTCGCTTAATAATATCCATT | TGCGACTGCTGCATGCA | S2 |
| *SlARF8* | TCGAATGCGGACGTTTACC | TAGCCTGAGTAACGTGCGATGT | S2 |
| *SlARF9* | CCAAGTTATCCTAATCTTCCTTCC | GTAAAGCCTCCTGGTCATATTTG | S2 |
| *SlARF10* | AACAGATAGGGAAAGAGGGACTTGAT | ATCCATTGACATTGCCAGGAA | S2 |
| *SlARF11* | AAATGTCCCTCATTTGGTTCTGTT | CCTTCAGCAGAGCCATCAGAAT | S2 |
| *SlARF12* | ATGGCGGAAGATGGTGAAAGT | AGAGAATGAAGCCAAAGAGATCACA | S2 |
| *SlARF13* | TGGACTCAGTAGCCCATCATTG | TGCCTGGCTCCCTGCAT | S2 |
| *SlARF14* | CCTGTTGGGACAAGATTTAAAATGA | CCATCTAAAGGGATCCATATCGC | S2 |
| *SlARF15* | CTCAAGCAGCAATATTAAAAGGAAATG | CCTGCCTGGTTTGATGAATCTAG | S2 |
| *SlARF16* | TGACACAGGCTGAAGTTTTGACG | GAAGGTAGAGGTAGCATAAGGCACA | S2 |
| *SlARF17* | TGAGTGGCGCTTCAGGCAC | CATCCCCTGCAACAAGCCTT | S2 |

S1. Wu, J., Peng, Z., Liu, S., He, Y., Cheng, L., Kong, F., et al. (2012). Genome-wide analysis of *Aux/IAA* gene family in Solanaceae species using tomato as a model. *Mol. Genet. Genomics*. 287, 295-311. doi: 10.1007/s00438-012-0675-y

S2. Kumar, R., Tyagi, A.K., Sharma, A.K. (2011). Genome-wide analysis of auxin response factor (ARF) gene family from tomato and analysis of their role in flower and fruit development. *Mol. Genet. Genomics*. 285, 245-260. doi: 10.1007/s00438-011-0602-7
